# Biocontrol potential and growth-promoting effect of endophytic fungus *Talaromyces muroii* SD1-4 against potato leaf spot disease caused by *Alternaria alternata*

**DOI:** 10.1186/s12866-024-03411-4

**Published:** 2024-07-09

**Authors:** Lihua Zhang, Wei Xu, Zhibo Zhao, Youhua Long, Rong Fan

**Affiliations:** College of Agriculture, Guizhou University, Guiyang, 550025 China

**Keywords:** Endophyte, *Talaromyces muroii*, *Alternaria alternata*, Potato leaf spot disease, Biocontrol

## Abstract

**Background:**

*Alternaria alternata* is the primary pathogen of potato leaf spot disease, resulting in significant potato yield losses globally. Endophytic microorganism-based biological control, especially using microorganisms from host plants, has emerged as a promising and eco-friendly approach for managing plant diseases. Therefore, this study aimed to isolate, identify and characterize the endophytic fungi from healthy potato leaves which had great antifungal activity to the potato leaf spot pathogen of *A. alternata* in vitro and in vivo.

**Results:**

An endophytic fungal strain SD1-4 was isolated from healthy potato leaves and was identified as *Talaromyces muroii* through morphological and sequencing analysis. The strain SD1-4 exhibited potent antifungal activity against the potato leaf spot pathogen *A. alternata* Lill, with a hyphal inhibition rate of 69.19%. Microscopic and scanning electron microscope observations revealed that the strain SD1-4 grew parallel to, coiled around, shrunk and deformed the mycelia of *A. alternata* Lill. Additionally, the enzyme activities of chitinase and β-1, 3-glucanase significantly increased in the hyphae of *A. alternata* Lill when co-cultured with the strain SD1-4, indicating severe impairment of the cell wall function of *A. alternata* Lill. Furthermore, the mycelial growth and conidial germination of *A. alternata* Lill were significantly suppressed by the aseptic filtrate of the strain SD1-4, with inhibition rates of 79.00% and 80.67%, respectively. Decrease of leaf spot disease index from 78.36 to 37.03 was also observed in potato plants treated with the strain SD1-4, along with the significantly increased plant growth characters including plant height, root length, fresh weight, dry weight, chlorophyll content and photosynthetic rate of potato seedlings.

**Conclusion:**

The endophyte fungus of *T. muroii* SD1-4 isolated from healthy potato leaves in the present study showed high biocontrol potential against potato leaf spot disease caused by *A. alternata* via direct parasitism or antifungal metabolites, and had positive roles in promoting potato plant growth.

## Background

Potato (*Solanum tuberosum* L.) is one of the four important economic and food crops, extensively cultivated worldwide [1]. However, with the rapid expansion of cultivation areas, the potato crop faces threats from various diseases, leading to significant yield losses. Among these, potato leaf spot, primarily caused by *Alternaria alternata*, is a prominent fungal disease [2]. *A. alternata* can not only reduce the yield but also the quality and safety of potato as the fungus can produce mycotoxins that may be detrimental to human and animal health [3, 4]. Therefore, it is essential to control leaf spot disease caused by *A. alternata* in potato production. At present, use of fungicides is the principal means to control plant fungal disease and their widespread use has led to growing problems like the emergence of new resistant mutant strains and toxic residues in food and the environment [5]. Biocontrol strategy, as an eco-agricultural way to control plant diseases, is much safer and a sustainable alternative to chemical agents and hence has attracted more attention in recent years [6–8].

Biocontrol efficacy is affected by several factors in practical applications, including light, temperature, humidity and the affinity of biocontrol microorganisms to plants. Among these factors, the affinity of biocontrol microorganisms to plants often contributes significantly to the instability of biocontrol agents in the field [9–13]. Therefore, the colonization ability of biocontrol microorganisms in plants is an important index to evaluate whether it has industrialization prospect [14]. Recently, endophytic microorganisms are gained more and more popularity due to their excellent colonization efficacy and better acclimatizing potential [15–18]. When colonized throughout plant tissues, endophytic microorganisms can provide systemic tolerance to many plant pathogens and as endophytes interact closely with the host plant, they show great potential to promote plant growth through phytohormone regulation, phosphate solubilization and iron carrier production [19–21]. The endophyte *Streptomyces sitangensis* 136 was found to not only inhibit the growth of *Sclerotium cepivorum* and reduce the incidence of white rot disease, but also significantly promoted the growth of garlic plants [22]. The fungal endophytes of *Trichoderma asperellum*, *Epicoccum nigrum* and *A. longipes* showed broad-spectrum antifungal activity against *Fusarium thapsinum*, *E. sorghinum*, *A. alternata* and *Curvularia lunata* and plant growth-promoting traits under in vitro and in vivo field conditions as well as effective colonization [23]. Therefore, use of endophytic microorganisms, especially isolated from host plants to improve the biocontrol efficacy is advocated and promising [24].

*Talaromyces muroii* belongs to *Talaromyces*, species of which are widely distributed in sponges, plants and soil and have great potential in agriculture, food, cosmetics, medicine and environmental protection [25]. In the field of agriculture, *Talaromyces* species can inhibit pathological changes in crops and promote crop growth [25]. The strain *T. muroii* EU18 inhibited the growth of *Colletotrichum coffeanum* causing coffee anthracnose and *C. musae* causing banana anthracnose effectively [26]. The cytochalasans (CYTs) from *T. muroii* sp. displayed a variety of biological activities such as antitumor, phytotoxins, virulence factors, antimicrobials and cytotoxins [27, 28]. Furthermore, *T. muroii* strain TM28, isolated from panax pseudoginseng roots, not only controls wheat *Fusarium* crown rot caused by *F. pseudograminearum* but also significantly enhances the fresh weight and height of wheat plants [29]. Therefore, *T. muroii* may have great biocontrol potential in disease prevention and plant growth promotion.

In the present study, endophytic fungi from healthy potato leaves were isolated aiming to screen antifungal activity strains to inhibit the pathogen of *A. alternata*, the causal agent of potato leaf spot disease. The isolate *T. muroii* SD1-4 was obtained after in vitro antifungal analysis of co-cultural incubation test, microscopic observation and activity determination of fungal cell wall chitinases and glucanases. Intriguingly, *T. muroii* SD1-4 strain also showed great potential on protecting potato plants from leaf spot disease and promoting the growth of potato seedlings in vivo. Results in the current study not only enriched the endophytic microbial resources for biological control of potato leaf spot disease but also provided a new insight into using endophytes for promoting plant growth.

## Results

### Screening and identification of endophytic fungus SD1-4

**Fig. 1:**
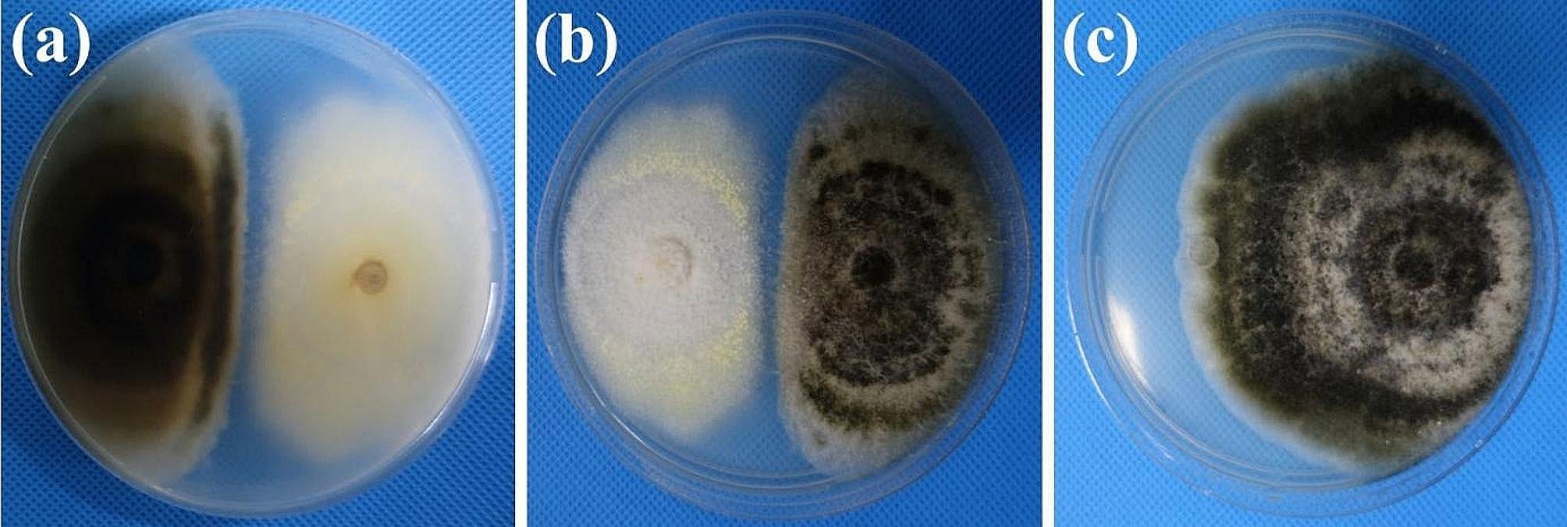
Co-cultures of *Talaromyces muroii* SD1-4 and *Alternaria alternata* Li11 on PDA plates for 7 days. Back view (**a**) and front view (**b**) of co-cultured colonies of the strain SD1-4 with light yellow color and *A. alternata* Li11 with dark brown color. (**c**) Colony of the strain *A. alternata* Li11

Six fungal strains were obtained from healthy potato leaves in Sandu. Among them, one fungus labeled SD1-4 showed strong inhibitory activity against *A. alternata* Li11 in vitro and the inhibition rate of mycelia growth was 69.19% in the dual culture experiment (Table 1; Fig. 1). So, the endophytic fungus SD1-4 was used in all subsequent experiments in this study.

**Fig. 2:**
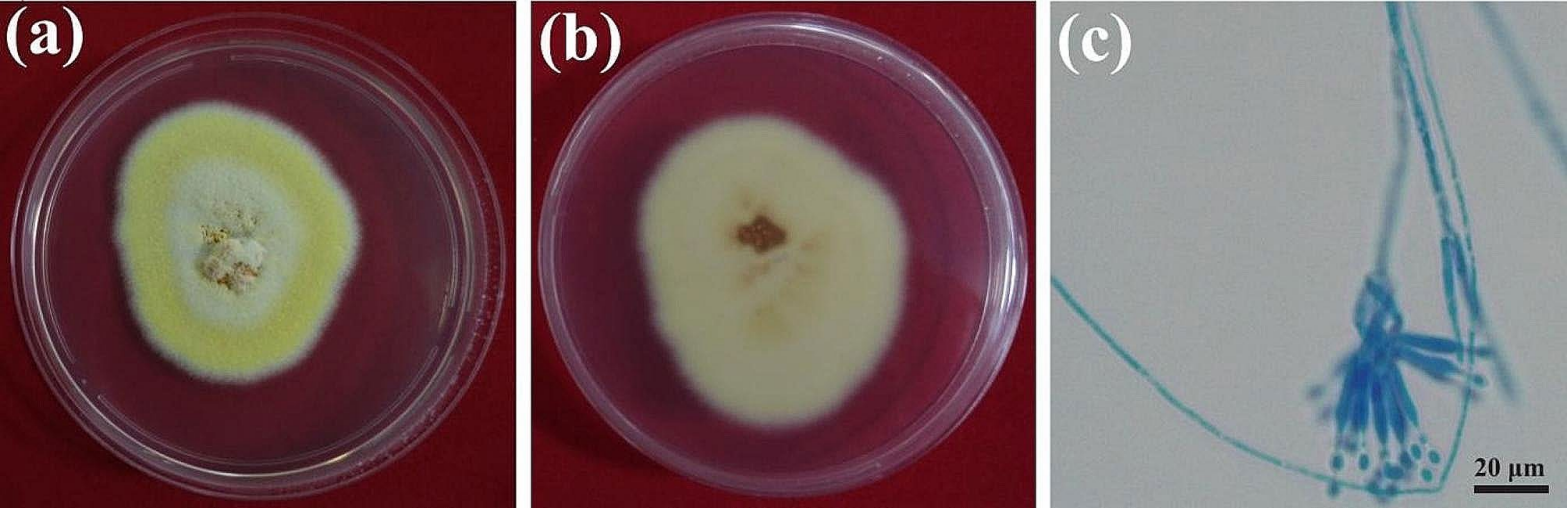
Morphology characterization of *Talaromyces muroii* SD1-4. Back view (**a**) and front view (**b**) of *T. muroii* SD1-4 grown on PDA media for 7 days. (**c**) Biverticillate conidiophores and long chains of conidia with lactophenol cotton blue staining

The strain SD1-4 was cultured on PDA media at 25ºC in dark for 7 days. The colony diameter was 47.55 ± 1.2 mm, with sparse yellow mycelia on the front of the colony and light-yellow color on the back of the colony. A large number of conidia were observed under a microscope. The conidium is short, smooth, single-or double-ringed, and conidiophores are smooth, oval to spindle-shaped, linear strung at the top of the phialides, with a size of 3–5 μm × 2–3.5 μm (Fig. 2).

**Fig. 3:**
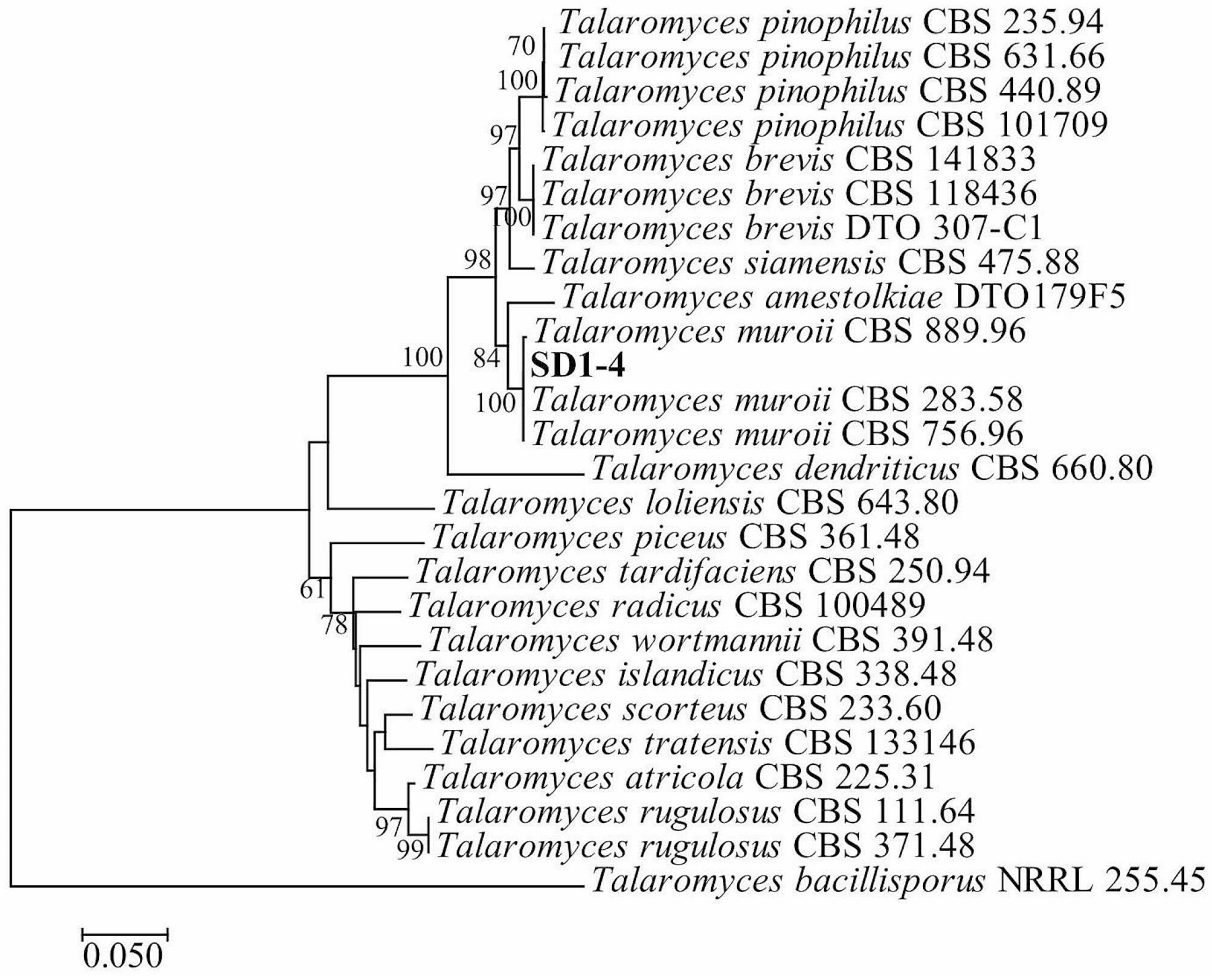
A phylogenetic tree based on ITS and *β-tubulin* sequences of *Talaromyces muroii* SD1-4 constructed using the maximum likelihood (ML) method in MEGA 7.0 software with bootstrap values based on 1000 replications

To identify the strain SD1-4, the ITS and *β-tubulin* gene were amplified by PCR and sequenced by Tsingke Biotech Co., Ltd. (Beijing, China). The similarity of the resulting nucleotide sequences was analyzed using the NCBI BLAST tool respectively. The ITS sequence of the strain SD1-4 exhibited a 99.58% identity with *T. muroii* (MK450747) and the *β-tubulin* gene sequence showed 99.45% identity with that of *T. muroii* (KM066151). A multigene phylogenetic tree was constructed using the maximum likelihood (ML) method in the MEGA 7.0 software. Phylogenetic analysis showed that the strain SD1-4 and *T. muroii* were clustered together, with approval rating of 100% (Fig. 3). Together with the morphological characteristics, the strain SD1-4 was identified as *T. muroii.*

**Table 1:** Inhibition rate of *Alternaria alternata* Lill by *Talaromyces muroii* SD1-4 after co-cultivation for 7 days

Treatment	Colony diameter of *A. alternata* (mm)*	Inhibition rate (%)
*A. alternata* Lill + SD1-4	17.00 ± 0.00b	69.19
Control	44.00 ± 0.17a	

*Numerical values were expressed as mean *±* standard error (SE) of triplicates. Different lowercase letters represented a significant difference (*p* < 0.05)

### Microexamination and scanning electron microscope observation

**Fig. 4:**
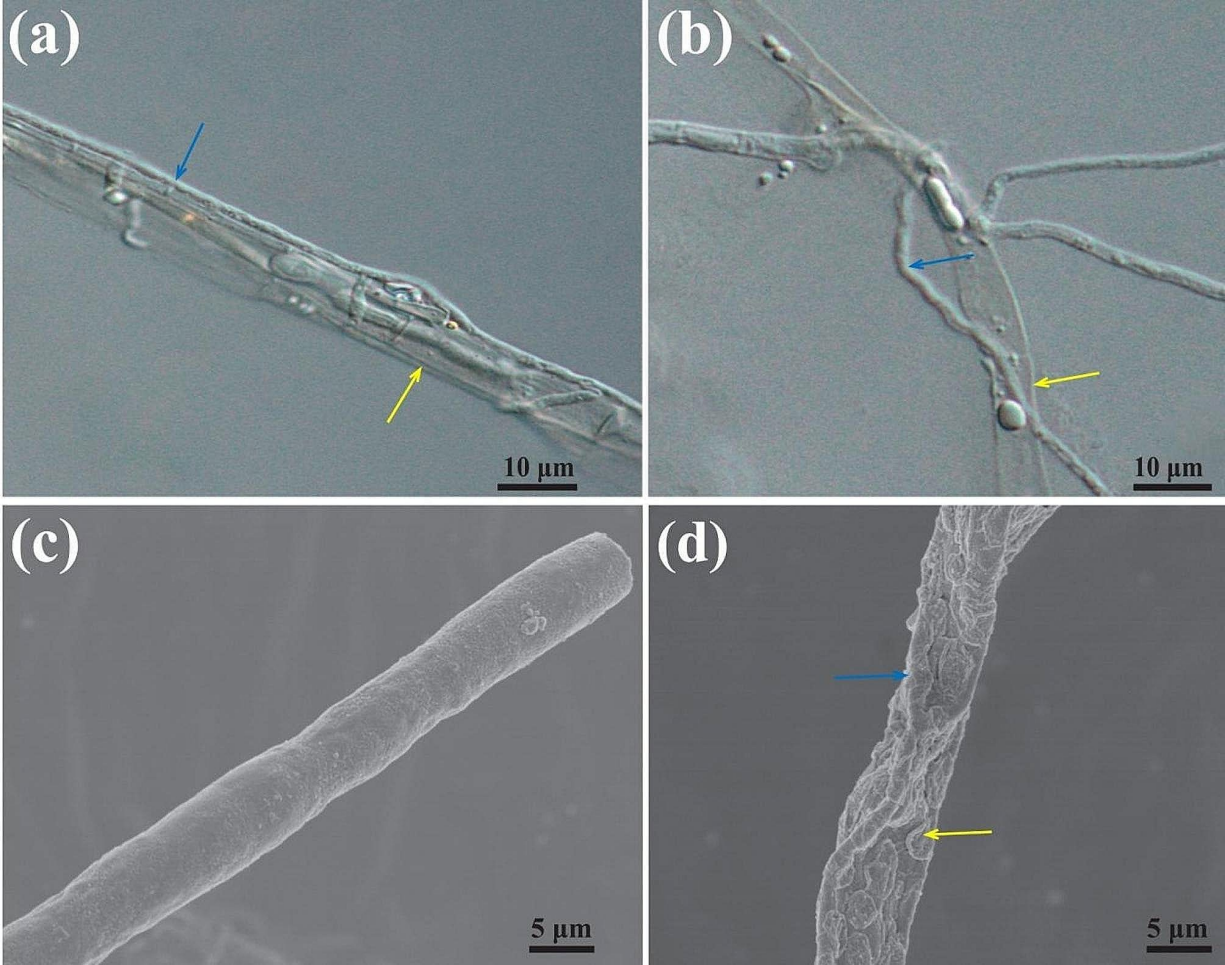
Micro-examination and scanning electron micrographs of mycelia of *Talaromyces muroii* SD1-4 in the co-cultures. (**a-b**) Optical morphology of *T. muroii* SD1-4 mycelia (blue arrow) and abnormal mycelia of *Alternaria alternata* Lill (yellow arrow) intertwined together. (**c**) Scanning electron micrographs of *A. alternata* Li11 mycelia and the co-cultures with the blue arrow represented for *T. muroii* SD1-4 mycelia and the yellow one for *A. alternata* Li11 mycelia (**d**)

Changes in the mycelial morphology of *A. alternata* Lill after *T. muroii* SD1-4 treatment were observed under optical microscopy that *T. muroii* SD1-4 grew in parallel with and coiled around the mycelia of *A. alternata* Li11, and caused mycelial deformation (Fig. 4a-b). Scanning electron microscope analysis further confirmed the antifungal action of *T. muroii* on *A. alternata* (Fig. 4c-d). Untreated *A. alternata* mycelia were smooth and full (Fig. 4c), while the co-cultured mycelia of *A. alternata* Lill were shrinkage and collapse with the mycelia of *T. muroii* SD1-4 parallel to or interwoven them (Fig. 4d). These results were consistent with the change in mycelial morphology observed under optical morphology, suggesting that mycelial parasitism would be an antibacterial mechanism by which *T. muroii* SD1-4 controled the *A. alternata* Lill.

### Chitinase and β-1, 3-glucanase enzyme activities

**Fig. 5:**
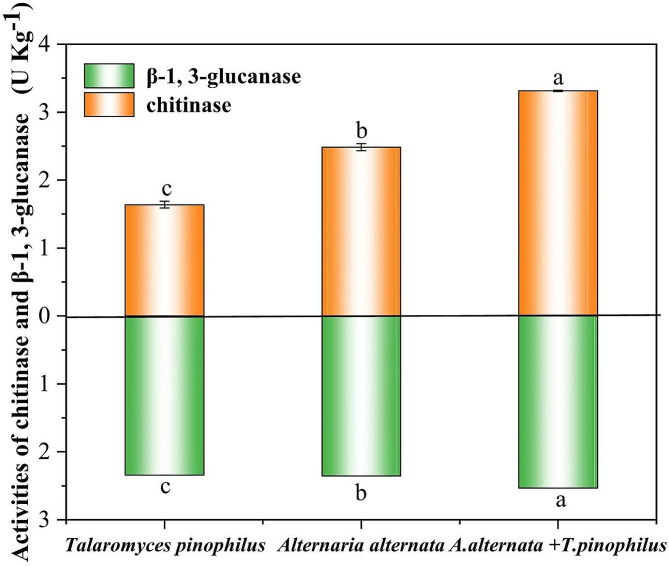
Chitinase and β-1, 3-glucanase activities of *Alternaria alternata* Li11 after treatment with *Talaromyces muroii* SD1-4. The different lowercase letters indicated significant difference between treatments (*p* < 0.05)

β-glucan and chitin are important components of fungal cell wall. Chitinase and β-1, 3-glucanase are important cell wall-degrading enzymes, and the activities of them can be evaluated the integrity of fungal cell wall. Compared with the individually cultured samples control, both the chitinase and β-1, 3-glucanase enzyme activities in the contact zone were significantly increased, suggesting that the mycelial cell wall of *A. alternata* Li11 maybe impaired after co-cultured with *T. muroii* SD1-4 (Fig. 5).

### Aseptic filtrate effects on the mycelial growth of *A. alternata*

**Fig. 6:**
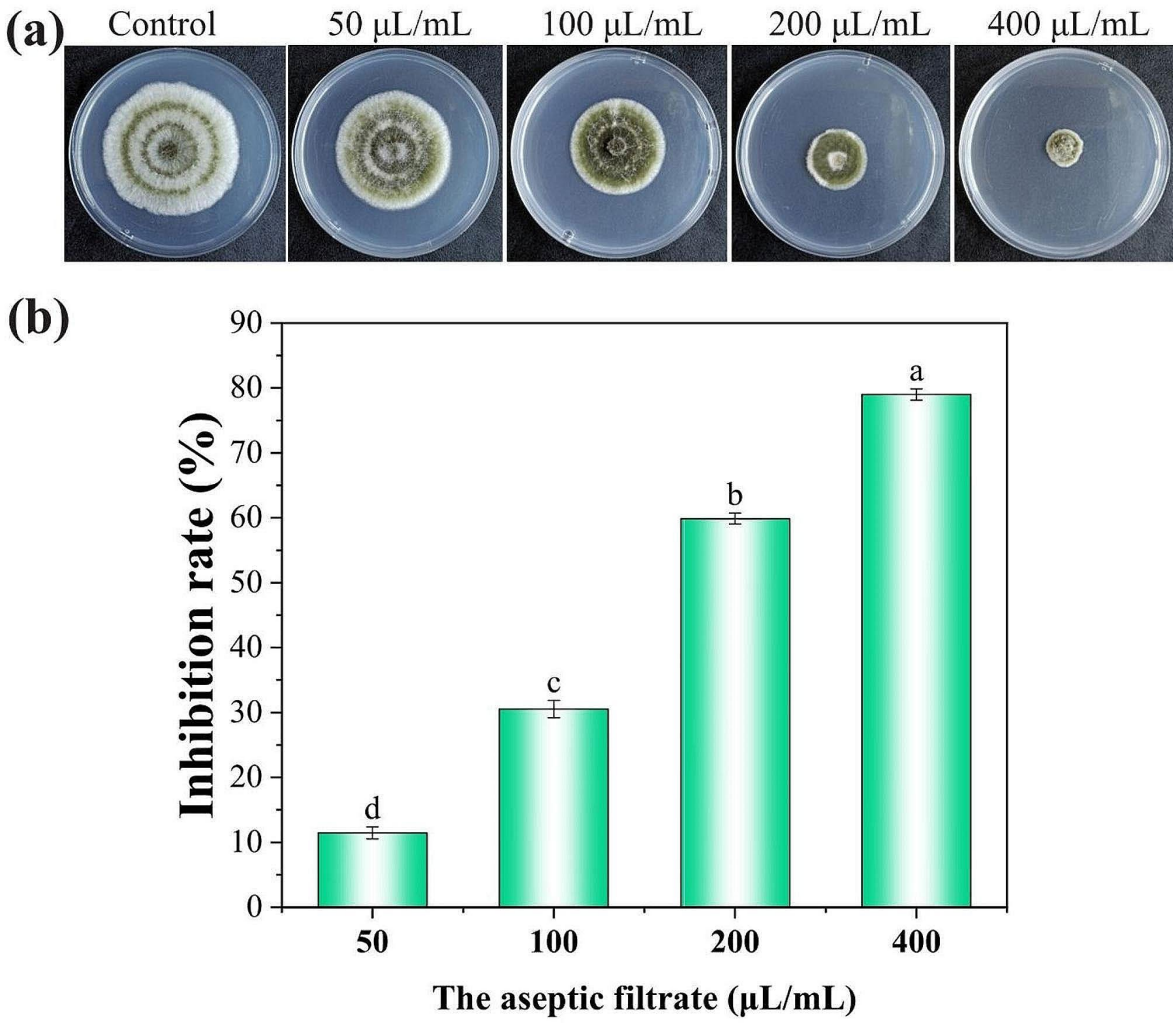
Aseptic filtrate effects of *Talaromyces muroii* SD1-4 on the mycelial growth of *Alternaria alternata* Lill. (**a**) Antifungal activity of different concentration aseptic filtrates against *A. alternata* Lill on PDA plates after 7 days. (**b**) Inhibition rate of different concentrations of aseptic filtrates against *A. alternata* Lill. The different lowercase letters indicated significant difference between treatments (*p* < 0.05)

To explore whether the secondary metabolites of *T. muroii* SD1-4 have antifungal activity, aseptic filtrate of *T. muroii* SD1-4 with different concentration were added to PDA plates to maintain the *A. alternata* Lill colony. Results showed that the colony diameter was significantly reduced along with the AF concentration increasing (Fig. 6a). The mycelial growth of *A. alternata* Lill was almost completely suppressed at the 400 µL/mL aseptic filtrate concentration, with an inhibition rate of 79.00% (Fig. 6b).

### Aseptic filtrate effects on the conidial germination of *A. alternata*

**Fig. 7:**
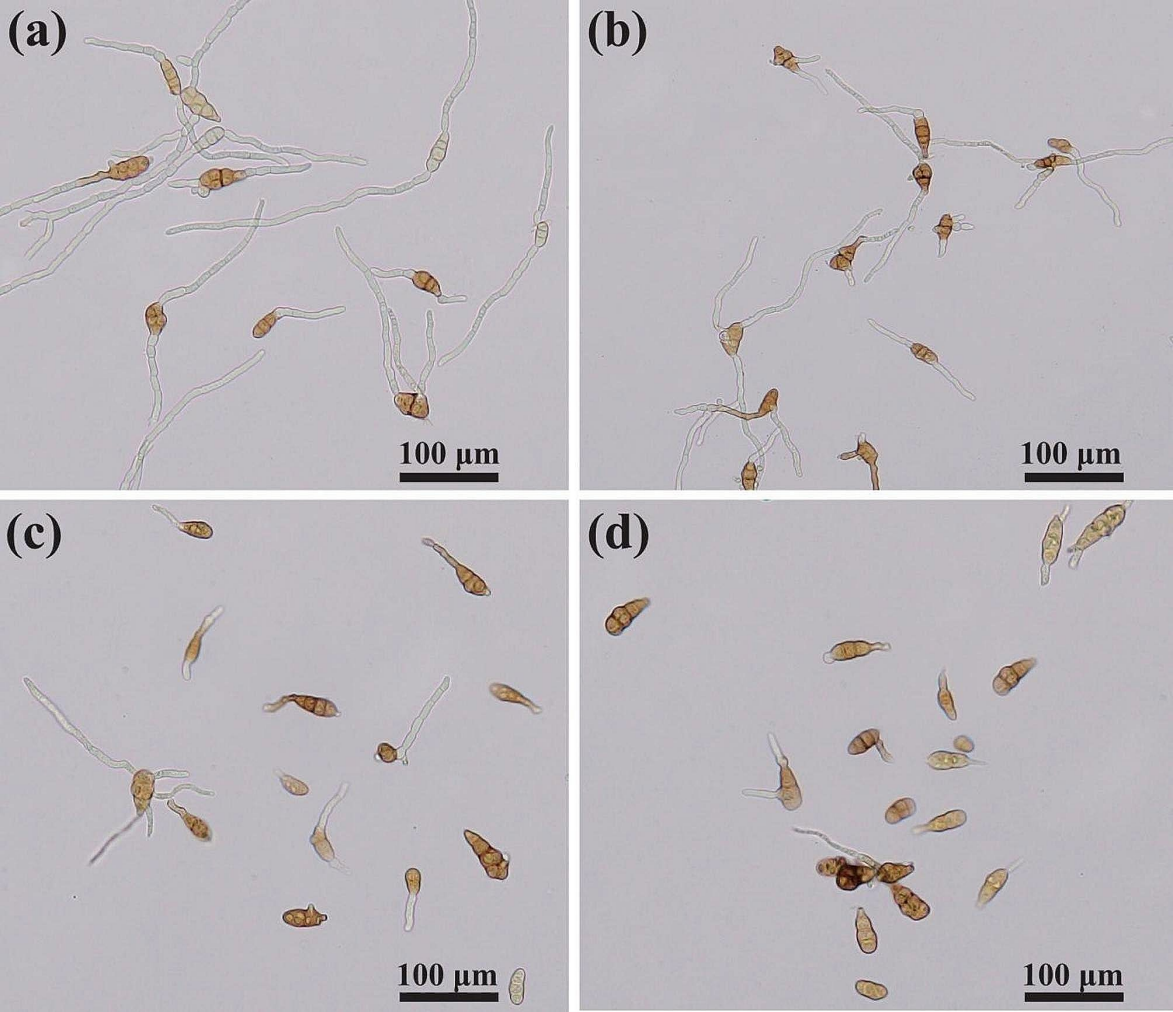
Determination of conidial germination of *Alternaria alternata* Lill treated with aseptic filtrates of *Talaromyces muroii* SD1-4. (**a**) Treated with sterile PDB filtrate as control. (**b**) Treated with 100 µL/mL aseptic filtrate of the strain SD1-4. (**c**) Treated with 200 µL/mL aseptic filtrate of the strain SD1-4. (**d**) Treated with 400 µL/mL aseptic filtrate of the strain SD1-4

Conidia are important reproductive structures for most pathogenic fungi in adverse environments and inhibition of conidial germination can reduce the reproduction and spread of fungi. As shown in Fig. 7, after treatment with aseptic filtrate, the conidia were deformed germinated and the germ tubes can not extend normally, which indicated the decrease in conidial infection ability. The conidial germination rate was 100.00% in the blank treatment without aseptic filtrate, whereas it was decreased to 50.00% or 19.33% under the treatment of aseptic filtrate with the concentration of 200 or 400 µL/mL, causing the inhibition rates of 50.00% and 80.67%, respectively (Table 2). These results indicated that conidia of *A. alternata* Lill were extremely sensitive to the aseptic filtrate of *T. muroii* SD1-4 in a concentration-dependent manner.

**Table 2:** Effect of aseptic filtrate of *Talaromyces muroii* SD1-4 on conidial germination of *Alternaria alternata* Lill

Treatment	Conidial germination rate (%)*	Inhibition rate (%)*
400 µL/mL AF	19.33 ± 0.67d	80.67 ± 0.67a
200 µL/mL AF	50.00 ± 1.15c	50.00 ± 1.15b
100 µL/mL AF	86.00 ± 1.15b	14.00 ± 1.15c
Control	100.00 ± 0.00a	—

*Numerical values were expressed as mean *±* standard error (SE) of triplicates. Different lowercase letters represented a significant difference (*p* < 0.05)

### Thermal stability of aseptic filtrate

**Fig. 8:**
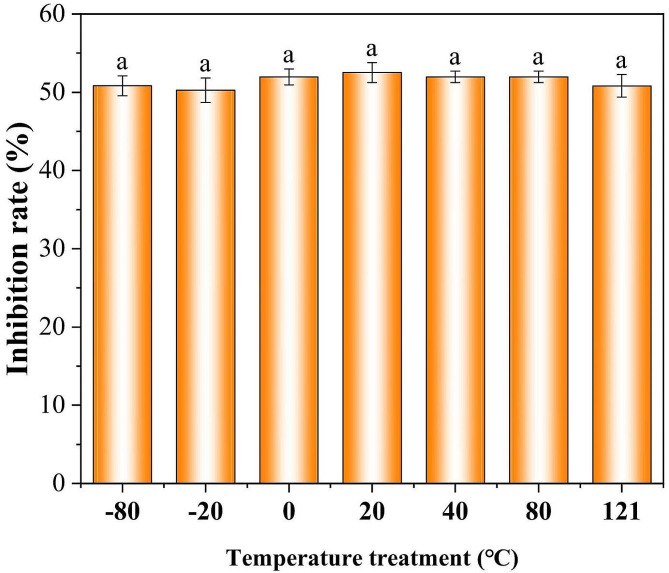
Stability of aseptic filtrate of *Talaromyces muroii* SD1-4 at 200 µL/mL treated with different temperatures. Numerical values represented mean ± SE of triplicates. The same lowercase letters indicated no significant difference between treatments (*p* ≥ 0.05)

The effects of different temperatures on the antifungal activities of the aseptic filtrate were evaluated and no significant changes were observed (Fig. 8), indicating that different temperature treatments had no effect on the antifungal components produced by *T. muroii* SD1-4. Thus, *T. muroii* SD1-4 had strong thermal stability.

### Pot experiments and assessment of the growth-promoting effect

**Fig. 9:**
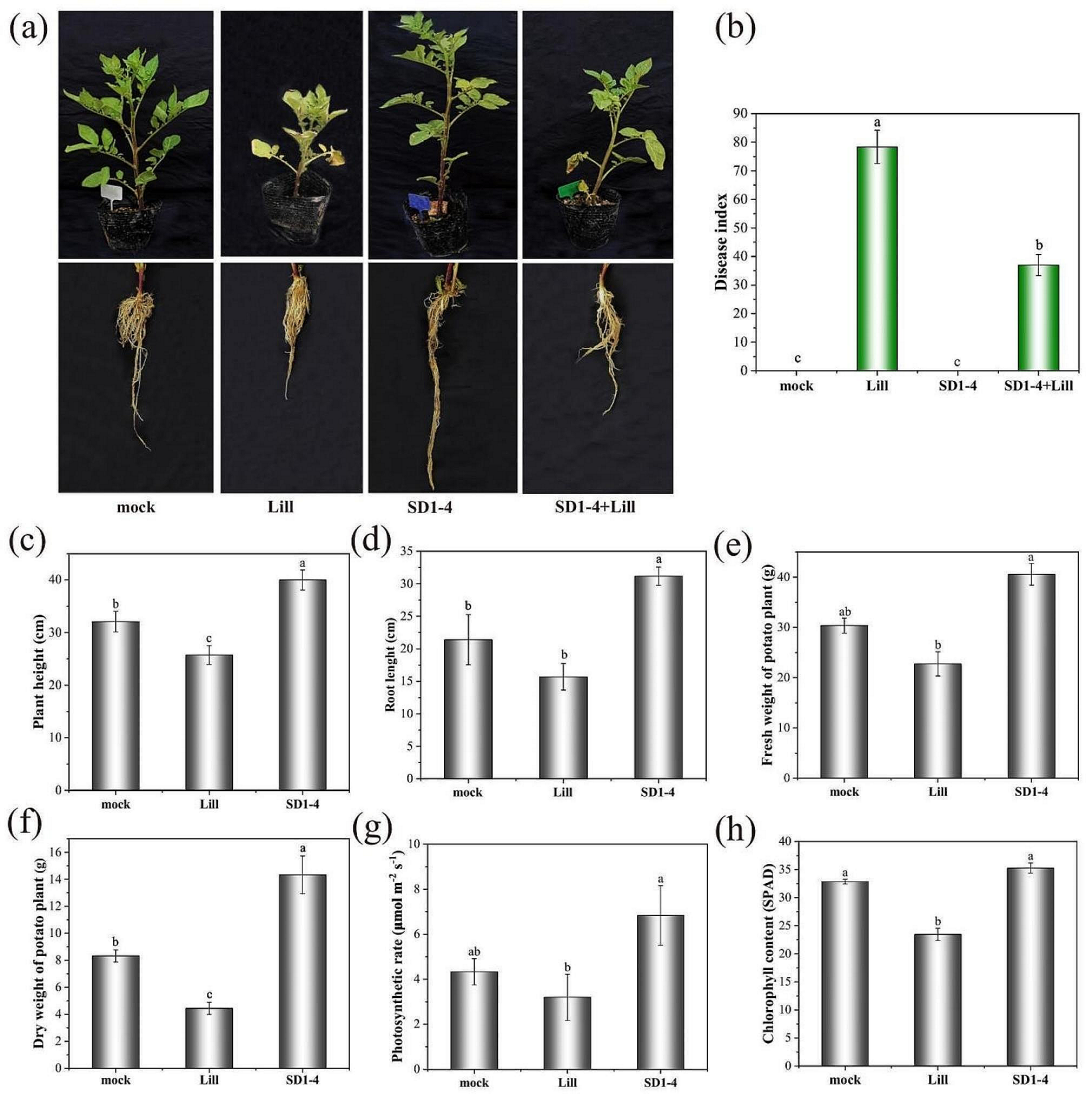
Effect of *Talaromyces muroii* SD1-4 and *Alternaria alternata* Lill on growth of potato plants. (**a**) Roots and phenotypes of potato plants treated with different strains or sterilized distilled water as mock after inoculation for 12 days. (**b**) Disease index of potato leaf spot disease. (**c-h**) Plant height, root length, fresh weight, dry weight, photosynthetic rate and chlorophyll content of potato plants treated with single strain of SD1-4, Li11 or mock. Bars with the different lowercase letters indicated significant difference between treatments (*p* < 0.05)

Results of the pot experiments showed the potent ability of *T. muroii* SD1-4 to reduce the disease severity and its biocontrol potential against potato leaf spot disease (Fig. 9a). At 12 days post inoculation, potato seedlings inoculated with the ddH_2_O (mock) or *T. muroii* SD1-4 only had no concentric brown spots while those inoculated with conidial suspension of *A. alternata* Lill showed typically leaf spot symptoms like brownish spots with concentric rings and chlorosis of potato leaves and the leaf spot disease index was 78.36 (Fig. 9b). However, the disease index of potato leaf spot via inoculating *T. muroii* SD1-4 for 2 days followed by conidia of *A. alternata* Lill was significantly reduced, which was 37.03 (Fig. 9b). These results indicated that the strain of *T. muroii* SD1-4 had the biocontrol potential to protect potato leaves from infection of *A. alternata* Lill.

In addition to the biocontrol effect of *T. muroii* SD1-4 on potato leaf spot disease, the plant growth promoting ability was also assessed by detection of the plant height, root length, fresh weight and dry weight of potato plants inoculated with the *T. muroii* SD1-4 only or mock after inoculation for 12 days. Results showed that above indicators of *T. muroii* SD1-4 treated potato plants was significantly increased by 24.73%, 45.65%, 33.67%, and 47.38%, respectively (Fig. 9c-f), suggesting that *T. muroii* SD1-4 had a pro-growth effect on plant morphology. Further analysis found that the chlorophyll content and photosynthetic rate of single *T. muroii* SD1-4 treated potato plants were highest, followed by the mock with 35.30 SPAD, 6.83 µmol·m^− 2^s^− 1^ and 32.87 SPAD, 4.33 µmol·m^− 2^s^− 1^ separately. In contrast, potato leaf inoculated with *A. alternata* Lill only had the lowest chlorophyll content and photosynthetic rate at 25.5 SPAD, 3.20 µmol·m^− 2^s^− 1^ (Fig. 9g-h), indicating that the chlorophyll synthesis and photosynthesis efficiency in *T. muroii* SD1-4 treated potato plants were significantly increased.

## Discussion

Potato leaf spot is a fungal disease on foliage that seriously affects potato production. Currently, chemical fungicides represent the primary method for disease management. However, given the goal of environmentally friendly disease control in agricultural settings, there is a continual need to develop novel approaches [30]. Biological control has become increasingly popular due to its advantages in pest, disease and weed management and ecological protection [6, 31–33]. A strong guarantee of safe crop production can be provided through biological control mechanisms, including parasitism and antibiotics [34]. In order to improve the colonization efficiency of biocontrol microorganisms in practical applications, use of endophytes from host plants in disease control is increasingly advocated [35]. Although an endophytic bacterial strain *B. velezensis* SEB1, isolated from the stem of *Vigna mungo*, showed good inhibitory activity on potato leaf spot pathogen of *A. alternata*, such as its reduced conidial germination and mycelial growth, isolation and screening endophytes with better antibiotic effect from the host potato leaf should be encouraged [36]. In the current study, the strain of endophytic fungus *T. muroii* SD1-4 was obtained, which exhibited good biocontrol effects on potato leaf spot disease as well as great potential of growth promotion on potato plants.

Cell wall integrity plays an important role in the normal growth and development of fungi and can improve fungal resistance to adverse environments [36]. In biological control, microorganisms tangle and penetrate the pathogens, resulting in abnormal perforation of the pathogenic cell wall, leakage of the cell contents, and thus the death of the fungus, which is a disease biological control principle known as parasitics [16, 37–39]. In this study, both optical microscopy and scanning electron microscope observation revealed that when *T. muroii* SD1-4 and *A. alternata* Lill were co-cultured, the mycelium of *A. alternata* Lill was entangled by *T. muroii* SD1-4, resulting in dried and crumpled deformities of the mycelium, so it was hypothesized that *A. alternata* Lill could be parasitized by *T. muroii* SD1-4. Subsequently, the enzyme activities of chitinase and β-1, 3-glucanase of mycelia collected from the co-cultivated area of *T. muroii* SD1-4 and *A. alternata* Lill or from the monoculture of both strains were conducted. Chitinase and β-1, 3-glucanase are considered to be the main components of fungal cell walls, and the rupture of fungal cell walls is often accompanied by an increase in the activities of chitinase and β-1, 3-glucanase enzymes [36]. The results of this study showed that chitinase and β-1, 3-glucanase activities of co-cultured mycelia were significantly increased than those of monoculture of both strains, suggesting the co-cultivation of *A. alternata* Lill and *T. muroii* SD1-4 may lead to the deformity and lysis of cell wall, suggesting that *T. muroii* SD1-4 could control potato leaf spot disease caused by *A. alternata* through parasitism. The parasitic mechanism employed by *T. muroii* SD1-4 in the present study was consistent with previously reported species in *Talaromyces*, such as *T. flavus* [40] and *T. pinophilus* [38].

Endophytic fungi may be used individually or in combination with different biocontrol mechanisms to inhibit plant diseases, among which production of antibiotics is an important mechanism in biological control [41]. Antibiotic action refers to the production of various antimicrobial compounds by biocontrol agents that inhibit or reduce the growth or proliferation of plant pathogens [41]. *Talaromyces* can produce many structurally diverse compounds with antagonistic properties, including esters, terpenes, steroids, alkaloids, polyketides, anthraquinones, glucanase, and others [25]. Hexane, ethyl acetate and methanol crude extracts of *T. muroii* EU18 at 1000 ppm inhibited the growth of colonies and sporulation of *C. coffeanum* [26]. The major bioactive compounds, eicosane, oleic acid, n-hexadecanoic acid, ethyl oleate, cis-vaccenic acid, heptacosane identified from *T. trachyspermus* have been reported to possess antioxidant, antibacterial and antifungal properties [42]. In the present study, the aseptic filtrate of *T. muroii* SD1-4 significantly suppressed the growth of *A. alternata* Lill and its mycelial growth and conidial germination was significantly inhibited by 79.00% and 80.67% respectively under the treatment of 400 µL/mL of the aseptic filtrate of *T. muroii* SD1-4, which may be due to some antimicrobial substances in the media produced by *T. muroii* SD1-4. Further study will focus on the identification of these antibiotic substances and confirmation of their biocontrol roles.

The efficacy of biocontrol agents can be affected by a variety of environmental factors resulting in reduced or increased antagonism [43]. Among these factors, temperature seems to be an important factor for *Talaromyces* strains, which are mostly thermophilic, and whether their secondary metabolites are also active at high temperatures [44], therefore thermal stability detection was performed on aseptic filtrates of *T. muroii* SD1-4 in the current study. Results showed that the aseptic filtrates can retain their stability between − 80ºC and 121ºC, which may provide an important implication for the utilization and storage of antibiotics of *T. muroii* SD1-4 in future.

Applying beneficial microorganisms as biocontrol agents is a promising and environmentally friendly method to protect plants against diseases and reduce the use of harmful chemicals [21, 30]. In the present study, *T. muroii* SD1-4, isolated from potato leaves, reduced the index of potato leaf spot disease by 41.33 when inoculated with the strain of SD1-4 compared with those only inoculated with *A. alternata* Li11. The decrease in the disease index may be due to the parasitism or antifungal metabolites of the strain SD1-4.

*Talaromyces* species can promote plant growth and improve crop quality through plant’s own action or production of various metabolites besides mitigating diseases [45–47]. The growth of cotton and potato can be promoted by *T. flavus* [48]. The antibiotic 3-O-methylfunicone, isolated from *T. pinophilus*, shows promising effects in protecting plants against pathogens and promoting plant growth [49]. The positive impact of *T. pinophilus* was observed through enhanced phosphorus uptake, leading to increased growth and yield of potatoes [47]. In the present study, compared with mock, the plant growth characteristics including plant height, root length, fresh weight, and dry weight of potato plants treated solely with *T. muroii* SD1-4 were significantly increased by 24.73%, 45.65%, 33.67%, and 45.65%, respectively. These results suggest that *T. muroii* SD1-4 has a positive pro-growth effect on morphological indicators. Green leaves are the main organs for photosynthesis, an important pathway for the synthesis of organic compounds, which plays a key role in plant growth. Photosynthetic pigments are essential for photosynthesis, and their content is closely related to the photosynthetic capacity of plants [50]. Hence, this study investigated the chlorophyll content and photosynthetic rate of potato leaves, revealing a significant increase following treatment with *T. muroii* SD1-4. The increase of photosynthetic parameters maybe benefited from the reduction of potato leaf spot disease or stimulated by potato plant own after application of *T. muroii* SD1-4. Anyway, the results of the present study provide evidence that the growth of potato plants can be promoted either indirectly or directly by the strain of *T. muroii* SD1-4.

## Conclusion

In this study, an endophytic fungus *T. muroii* SD1-4 was isolated from healthy potato leaves and evaluated as a promising biocontrol agent for potato leaf spot disease caused by *A. alternata*. The strain SD1-4 showed typical parasitism and antifungal activity against the pathogen of *A. alternata* Li11 *in intro* and exhibited great potential for protecting potato plants from leaf spot disease and promoting the growth of potato seedlings in vivo. These results provided valuable insights into utilization of endophytic fungi to bio-control potato leaf spot disease and enhance growth of potato plants.

## Materials and methods

### Pathogen and endophytic fungal strains

The pathogen *A. alternata* Li11 was isolated from potato leaves showed typical leaf spot lesions and the endophytic fungi strain SD1-4 was isolated from potato healthy leaves. Both of the potato leaf samples were collected from potato fields at San du county (26°02′N, 107°52′E), Guizhou province, China, in 2022. The healthy leaves were properly washed with ddH_2_O, and then cut into small pieces (ca. 5 mm) in 1.5% NaClO surface sterilisation for 45 s followed with triple washes using ddH_2_O, and dried with sterilized filter paper. Finally, these leaves were placed on PDA plates and cultured in the dark at 25 °C for 7–14 days until the ideal fungal growth was achieved [51]. The pure colonies were soaked in 25% glycerol and stored at − 80ºC until use.

### Endophytic fungi screening

The isolated strains were tested in vitro for their antifungal effect against *A. alternata* Lill as reported method per [38] with slight modifications. In brief, a 5-mm-diameter disk from the margin of each actively growing colony of *A. alternata* Lill was transferred to PDA plate. Then, a disk of the isolated strains was inoculated at the symmetrical points at 25 mm on the other side of the pathogen. Control tests were also performed using the pathogen alone. All plates were incubated for 7 days at 25ºC in darkness. The inhibition rate was calculated using the formula: Inhibition rate (%) = (R1-R2)/(R1-5) × 100, where R1 and R2 was the radial growth of *A. alternata* Lill on the control and dual culture plate, respectively. All experiments were performed in triplicate and the means were used.

### Identification of the endophyte strain SD1-4

The morphologies of the mycelia and conidia of strain SD1-4 incubated at 25ºC for 7 days were observed under an optical microscope (LEICA ICC50 W, Leica Microsystems Co., Ltd., Shanghai, China) after staining with lactophenol-cotton blue and identification of fungi was based on previous method [26].

In addition, the sequences of ITS region and *β-tubulin* of the strain SD1-4 were amplified by PCR [52]. After sequencing and alignment of these sequences, a multigene phylogenetic tree was constructed using the maximum likelihood (ML) method in MEGA7.0 software with bootstrap values based on 1000 replications. The sequences of ITS and *β-tubulin* obtained in this study were deposited under GenBank accession numbers OR835849 and OR854814, respectively.

### Micro-examination of the co-cultures

The interaction between the strain SD1-4 and *A. alternata* Lill was examined using the slide culture method [53]. After incubation at 25ºC and 75% humidity until the colonies overlapped, the mycelia in overlapping areas or in single incubations was respectively selected for optical microscope (digital microscope system Keyence VHX-7000) and scanning electron microscope (SU-8010, Hitachi, Tokyo, Japan) observation at 3.0 kV at 10,000× of magnification [51, 54].

### Detection of chitinase and β-1, 3-glucanase activities

To assess the effect of strain SD1-4 on the cell wall of the strain *A. alternata* Lill, chitinase and β-1, 3-glucanase activities of the strain SD1-4 or *A. alternata* Lill in single cultures and in overlapping areas of co-cultures were determined respectively using commercially available kits (Beijing Solarbio Science & Technology Co., Ltd., Beijing, China) following the manufacturers’ instructions. All experiments were performed in triplicate.

### Antifungal activity of the aseptic filtrate of the strain SD1-4

Aseptic filtrate was collected by filtration of the liquid PDB cultures of SD1-4 at 25ºC for 5 days through a sterilized filter gauze and a 0.22 μm membrane filter, and then its efficacy against *A.alternata* Lill was assessed according to the previous study [51]. The pathogen disks were inoculated on the center of PDA plates containing 50 µL/mL, 100 µL/mL, 200 µL/mL and 400 µL/mL (v/v) aseptic filtrate, respectively. After incubation for 7 days at 25ºC, the colony diameters were measured in two perpendicular directions. The treatment using sterilized PDB filtrate was set as control. All experiments were repeated for three times. The inhibition rate was calculated as follows: Inhibition rate (%) = [(Dc-Dt)/(Dc-5)] × 100, where Dc and Dt represents the colony diameter of the strain *A. alternata* Lill on the control and under the treatment of different aseptic filtrate concentrations.

### Aseptic filtrate effects on the conidial germination of *A. alternata*

The conidial germination of *A. alternata* Lill was detected after treatment with the aseptic filtrate of strain SD1-4. In brief, the aseptic filtrate and conidial suspension of *A. alternata* Lill were mixed in a 1: 1 (v: v) ratio so that the final concentrations of aseptic filtrate were 100 µL/mL, 200 µL/mL and 400 µL/mL, respectively, and the conidial concentration was 1.0 × 10^7^ conidia/mL [51, 55]. Then, 20 µL mixture was dripped onto the center of a concave slide and covered with a cover slip. The mixture was incubated in a petri dish with moist filter paper at 25ºC for 12 h and conidial germination was tested under an optical microscope. Conidial germination was judged on the basis that the length of the shoot tube was more than half the length of the conidia. Three fields of view of each treatment with at least 50 conidia in each field of view were used. The experiment was performed in triplicate and the treatment of the liquid PDB was used as a control.

### Thermal stability of the aseptic filtrate

The stability of the aseptic filtrate was evaluated under the treatment of different thermal conditions as previously described with minor modifications [51]. The aseptic filtrate was treated at − 80ºC, − 20ºC, 0ºC, 20ºC, 40ºC, 80ºC and 121ºC for 30 min respectively, and then returned to room temperature. After the different treatments, the effects of 200 µL/mL aseptic filtrate on the inhibition of mycelial growth of *A. alternata* Lill were determined to evaluate the filtrate stability. All experiments were performed in triplicate.

### Pot experiments and assessment of the growth-promoting effect

Protective effect of the strain SD1-4 against potato leaf spot disease and its growth-promoting effect were investigated in pot experiments. The conidial suspensions of strain SD1-4 and *A. alternata* Lill were collected by incubation in liquid PDB at 25℃, 150 rpm for 5 days, respectively. Conidia were harvested by centrifuging at 5000 g for 1 min and suspending in ddH_2_O to the concentration of 1.0 × 10^7^conidia/mL [55].

Uniformly grown healthy potato seedlings at the 5-leaf-stage were used for the pot experiments. The 5-leaf-stage potato seedlings were treated by foliar spraying with 30 mL of ddH_2_O (mock), 30 mL of the conidial suspension of the strain SD1-4, 30 mL of the conidial suspension of *A. alternata* Lill, and 30 mL of the conidial suspension of the strain SD1-4 sprayed 2 days before 30 mL of the *A. alternata* Lill conidial suspension, respectively [30, 56]. All the potato plants were kept in a greenhouse at 25℃, 16 h light: 8 h dark and 80% relative humidity. Six plants were employed in each treatment and the pot experiment was repeated for three times.

Disease severity of each seedling was recorded at 12 days following nine categories [30]. Disease index was calculated according to the formula: Disease index= (Sum of all disease rating/Total number of ratings × Maximum disease grade) × 100 [57]. In addition, to evaluate the influence of the treatment on growth index and physiological index of potato seedlings, the plant height (cm), root length (cm), fresh weight of potato plant (g), dry weight of potato plant (g), photosynthetic rate (µmol·m^− 2^s^− 1^) and chlorophyll content (SPAD) of potato plants were measured following the published method [58].

### Statistical analyses

Data was analyzed using the one-way ANOVA performed as per LSD multiple range test to determine the significant difference at *p* < 0.05. Charts were plotted with Origin 2021. Statistical analyses were performed using Excel 2010 and SPSS v. 23.0 software.

## Data Availability

Sequence data that support the findings of this study have been deposited in the NCBI Nucleotide database with the GenBank accession numbers of OR835849 and OR854814.

## Abbreviations

PDA: Potato Dextrose Agar (Medium)
PDB: Potato Dextrose Broth
ANOVA: Analysis of Variance
ddH_2_O: Sterilized Distilled Water
LSD: Least Significant Difference
